# Caught between is and ought: The Moral Dissonance Model

**DOI:** 10.3389/fpsyt.2022.906231

**Published:** 2022-12-23

**Authors:** Hans Te Brake, Bart Nauta

**Affiliations:** ^1^ARQ Centre of Expertise for the Impact of Disasters and Crisis, Diemen, Netherlands; ^2^ARQ Centre of Expertise on War, Persecution and Violence, Diemen, Netherlands

**Keywords:** moral injury, moral dissonance, decision-making, moral distress, military, social context, modeling, framework

## Abstract

Considerable academic effort has been invested in explaining the causes of, and processes behind moral injury. These efforts are mostly focused on assessment and treatment within a clinical setting. Collective and social factors contributing to moral injury are often overlooked in current literature. This perspective article considers the role of contextual factors associated with moral injury and proposes a framework that describes their relation to individual aspects. The resulting Moral Dissonance Model (MDM) draws on existing theories and frameworks. The MDM explains how dissonance can occur when the actual behavior–the response to a morally challenging situation–contradicts with morally desirable behavior. Individual and collective factors, which change over time, contribute to the experience of dissonance. The inability to sufficiently solve dissonance can lead to moral injury, but not as a matter of course. The MDM can help to understand the underlying processes of moral distress. It raises awareness of the influence of public debate and controversy, and the resulting changing societal attitudes over time. Its implications and future use are discussed.

## 1. Introduction

Moral injury describes the suffering that may develop after a violation of deeply held moral beliefs and values. While consensus on the definition of moral injury is currently lacking (1), moral injury is generally assumed to result from the betrayal by a leader or trusted authority (2) or exposure to events that involve either perpetrating or witnessing actions that violate one's core beliefs (3).

The concept of moral injury is rooted in discontent with dominant theory and treatment regarding post-traumatic stress disorder (PTSD). Although PTSD and moral injury show overlap in their respective symptomatology (2, 4), the moral and social dimensions of military distress were believed to be lacking in the current definition of PTSD (3–7). Thus, around a decade ago, military psychiatrists and psychologists adapted the concept moral injury to capture moral conflict-colored feelings of shame, guilt, betrayal and anger as a result of soldiers' deployment (5).

Most current studies on moral injury focus on its clinical assessment [e.g., (1, 8–11)]. As noted by Molendijk et al. (5), a consequence of such a focus is that moral injury is turned “…into an individual-focused and pathologizing construct which explains trauma only in terms of intra-psychic and inter-personal processes, and gives sufferers the status of patients with mental disorders” (p. 3). This might lead to unnecessarily pathologizing of what can be considered normal moral processes (12).

In addition, by focusing on individual suffering the role of contextual factors is easily overlooked. Given the fact that morality itself is inherently social, it is unwarranted to treat moral injury as a concept that only relates to the experience of an individual (13–17). Contextual factors are all circumstances surrounding the individual's experience of an event, and include (military) culture, political mandate, and societal attitudes. These circumstances contribute to the occurrence of moral distress [e.g., (1, 5)]. For instance, Molendijk and colleagues (18, 19) describe how moral distress results from political decision-making and framing, but also from a lack of societal recognition (e.g., criticism and/or misplaced admiration regarding military missions).

In this perspective paper, we explore how to conceptualize the individual experience of moral distress in interaction with contextual factors, which can change over time. In doing so, we attempt to describe the manifestation of moral distress as a normal process, and move away from the focus on individual suffering and treatment of most current research. Building upon established theories and frameworks, we propose the Moral Dissonance Model (MDM) as a visualization of the continuous interplay between individual experience and contextual aspects. We believe that such a model is applicable to a wider context than the military, a research direction advocated by the reviews of Molendijk et al. (5) and Griffin et al. (1).

## 2. Establishing a comprehensive framework around is and ought

### 2.1. Distinguishing actual behavior from its consequences: The is

Litz et al. (3) describe a conceptualization of moral injury that is highly influential in current literature [e.g., (11)]. Their model starts with the occurrence of a “morally injurious experience”–as noted by Farnsworth et al. (14), this sometimes is even reduced to simply “moral injury.” Such terms confound *the occurrence* of a specific behavior (or lack thereof) with *a specific outcome* of that behavior (e.g., moral injury), which may “contribute to tautological assumptions about the impact of these events (e.g., that certain events necessarily cause moral injury)” [(8), p. 2]. As a first step in deconstructing moral distress, we believe it is important to steer away from such assumptions.

In the model by Litz et al. (3), transgression (i.e., the experienced dissonance between an individual's morals and their actual behavior in reaction to a morally distressing event) is the starting point of a path that leads to moral injury. This framework focusses on the individual consequences of a confrontation with a morally difficult situation and it does not take the broader surrounding context into account. Social factors–which are part of the context–are included in models such as proposed by Koenig et al. (20) and the dual process model of moral injury (21). However, these models use context primarily in relation to morally traumatized individuals within their social environment, i.e., social alienation, social anxiety and social isolation are mentioned as the consequences of traumatization (1, 21).

Models describing how people make sense of, judge, and make decisions in morally colored situations can be found outside clinical literature. These models describe the processes that influence behavior before and during a morally difficult situation, instead of focusing on its consequences. For instance, organizational pressures, moral norms and behavior expected from others are some of the factors mentioned in the Ethical Dissonance Cycle (22), the Integrated Ethical Decision-making Model (23) and the model of moral choice behavior (24).

We have applied this contextual strand of thought using the Sensemaking Intuition Model (25). Sonenshein describes how the individual, confronted with an ethical issue, constructs an instantaneous intuitive judgement, i.e., an automatic affective reaction such as “right” or “wrong.” This sensemaking is shaped by collective and individual factors; morality and ethics develop in childhood and are influenced during lifetime–especially within organizations such as the military or police where recruits are immersed in a new moral system (26, 27). Collective factors (e.g., the expectation of others and existing moral norms) also play a role in making morally challenging decisions (24).

Intuitive judgements and the resulting behavior occur rapidly and often without awareness. The actual behavior or response now constitutes the *is*: it cannot be altered and is (historically) situated in a certain place and time. A non-response or ‘freezing' response can be considered a reaction too; individuals feel responsible for the behavior shown even if they were unable to act in a given situation and bear no moral responsibility for harm (28, 29).

### 2.2. Justifying behavior: The ought

Directly following the shown behavior, the individual needs to rationalize and justify it toward him/herself and others. This happens *post-hoc*, and it is in this phase that dissonance can occur when the actual behavior contradicts with a morally more desirable behavior; a sense of *ought*. People experience dissonance as problematic and are intrinsically motivated to reduce its consequent psychological stress (30, 31).

In daily life, most of us can adequately deal with experienced dissonance. This can be partly explained by the individual's cognitive flexibility. Cognitively flexible people perceive difficult situations as controllable, are able to perceive multiple alternative explanations for life occurrences and human behavior, and are able to generate multiple alternative rationalizations to justify behavior (32, 33). Proneness to feelings of shame and neuroticism are two other aspects that may cause a higher susceptibility to experience dissonance (3). These individual factors influence the way somebody perceives and internally experiences a morally distressing event.

The distinction between what is and what ought-to-be can be traced back to 1739 when it was mentioned by Hume–albeit in a different manner (34). Hume believed it to be inherently impossible to deduce a (prescriptive) ought-statement about moral values from a (descriptive) is-statement on the state of affairs in the world. He thus separated the world of facts from the world of morality: the so-called Hume's Guillotine. While we, unlike Hume, do not purport to offer views on moral epistemology, the analogy of the is-ought problem can be made to the experience of moral dissonance. The distinction between descriptive and prescriptive cognitions has been used in the study of moral injury. Indeed, Farnsworth (35) proposed that moral injury is defined in part by prescriptive cognitions–that is, a person's judgement about what morally ought to be. For example, a veteran may feel guilt that he did not rescue a fellow soldier trapped in enemy fire and scolds himself as a coward. The veteran, in effect, prescriptively states that he should have acted differently (35).

### 2.3. The Moral Dissonance Model: Changes through context and time

**Figure 1:**
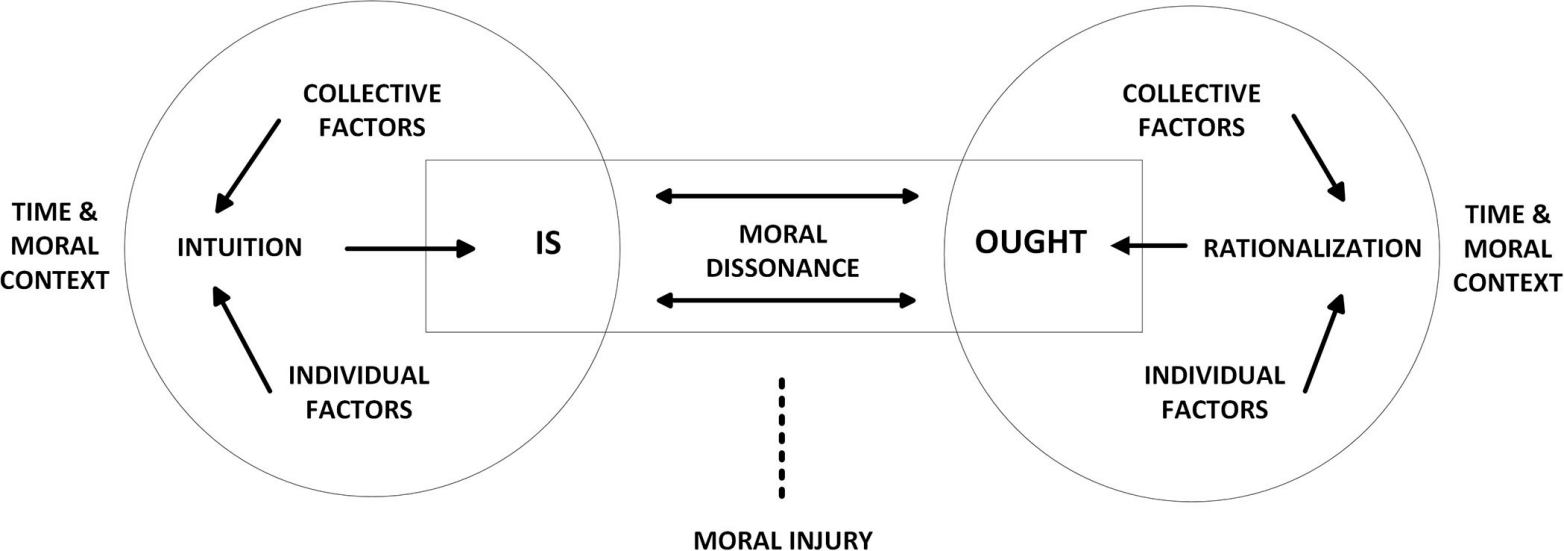
The Moral Dissonance Model.

The MDM combines the two elements described above and is depicted in Figure 1. Confronted with a morally ambiguous situation the individual intuitively tries to make sense of it before responding. This initial reaction constitutes the objective/actual behavior, or the non-alterable *is*. This is shown in the left of the Figure 1. After the initial response people will try to rationalize their behavior to themselves (individually) and others (socially), depicted on the right of the Figure 1. Moral dissonance arises when the displayed behavior is experienced to conflict with a morally more desirable behavior (*ought)*, shown in the middle of the Figure 1. Simply put, an individual will think: “I should have acted otherwise”. An enduring inability to reach consonance can lead to moral injury (as depicted by the dotted line in the Figure 1).

The dissonance between is and ought can become more pressing by changing moral contexts-as there is a continuous change between the displayed behavior and a present, ever-changing sense of how the individual should have acted differently. What is striking about the stories of persons who experienced morally difficult situations, is their lively recollection about the events—years, sometimes decennia, after they occurred (36). These experiences, historically situated in a certain context, such as war, are often very different from their present-day lifeworld. The is-ought dynamic is susceptible to such changes.

For one, people personally develop over time and can develop new moral values, also depending on the changing social contexts in which one lives and works. Accordingly, a person can re-alter the idea of how he or she ought to have acted in the past. Judgements by society, organizations, family, friends, or bystanders can also upset an earlier felt balance (18). In certain moral contexts expectations prevail that allow or even encourage actions that are vehemently rejected in other contexts. For instance, there can be a stark difference between the circumstances and moral values of the workplace and those in the private sphere; the use of violence in the military is seen as morally acceptable and can conflict with privately held values. After acting out (violently) in concurrence with military values, privately a person can think that he or she ought to have acted differently, causing dissonance.

Contexts change not only over place but also over time, for example when a service member returns home after deployment of several months. During their reintegration into society, unwarranted admiration for veterans, or, conversely, public criticism on a military mission can result in the experience of misrecognition and may cause moral injury (18). The moral world of the military mission wherein a service member acted in a certain manner, then collides with the moral world of society.

As an illustration of how a change of context can influence the individual's perception of a morally difficult situation, we describe the experience of a Dutch veteran stationed in Afghanistan^1^. As part of the International Assistance Security Forces (ISAF), the veteran regularly had to visit with an Afghan police commander, as he was an important actor in local security and in the ISAF network. This commander was accompanied by a so-called “chaiboy,” a 10-year-old boy who danced and poured tea for the guests. It was known that the commander also sexually abused the child. The veteran explained how at the time, the situation caused dissonance: despite feeling extremely uncomfortable, the veteran chose not to intervene. She knew that it was impossible—as a woman and a foreigner—to confront the commander. It would ruin the relationship.

Her rationalization was sufficient in the context of the mission in Afghanistan. Upon return to the Netherlands, however, following the pregnancy with her first child, doubts did arise. With the birth of her daughter, she realized how vulnerable children are. Many years later, through individual change and a change of context, she felt powerless–an emotion she could not allow before. It was impossible to change the outcome of the Afghanistan dilemma, she did however look for positive changes she could make in her everyday life, by speaking out against discrimination or sexual intimidation, and by deciding, three weeks after the birth of her first daughter, to have her second daughter adopted.

## 3. Discussion

This perspective paper addresses the issue that current moral injury literature is overly focused on a clinical construction of the concept–where, in fact, moral injury has roots in, and implications for, both individual experiences and the social fabric itself (17). We introduced the Moral Dissonance Model (MDM) as a conceptual framework which can help understand the interplay of individual and collective factors related to moral injury beyond the clinical setting. Although the MDM relates to the influential causal framework of Litz et al. (3), the latter model specifically aims to reconstruct a process that leads to moral injury. The MDM, on the other hand, takes an opposite approach: dissonance, which can occur in everyday situations, is a normal human reaction that will not necessarily end in “injury”–but still can be tremendously distressing (37, 38).

In constructing the MDM, we shy away from the more clinical reasoning about moral injury. As noted by Griffin et al. [(1), p. 357], moral distress “… is a product of culturally imbued, shared values that are internalized by individuals—some of which (e.g., loyalty to country) may conflict with others (e.g., thou shalt not kill).” Even if moral injury occurs, it is not solely a product of intrapsychic conflict, and recovery is intrinsically connected with the extent personal views are shared with others. The context (be it family, community, working organization or culture) is part of the healing process in which the individual must return (p. 358). Such a general process of dissonance is also applicable to work sectors outside the military domain, which often is a focus in moral injury research (2, 39). During the recent COVID-19 pandemic, it was obvious how much medical professionals were confronted with moral dilemmas (40, 41). Indeed, the term moral distress relates to the nursing profession (42) and implies the experience of knowing the right thing to do while being in a situation in which it is nearly impossible to do so.

We believe the MDM can help understand the underlying processes of moral distress and put them into words–it raises awareness of the influence of public debate and controversy, and the resulting changing societal attitudes over time. However, we do recognize the MDM has limitations. A generalized model does no justice to the complex reality people in moral ambiguous situations are confronted with. Our example of the Dutch veteran stationed in Afghanistan is a case in point: she experienced dissonance from the outset and this was not completely resolved with her *post-hoc* rationalization. Dissonance caused by a moral violation, even before the actor has shown any behavior, is not explained by the MDM. Also, the MDM does not explain consequences as described by McDonald (43), who holds that moral injury does not only concern one's sense of moral failing, but also the painful thought that moral structure does not exist in the world at all.

In “normalizing” the moral dissonance process, also new questions emerge. In what way differs immediate dissonance caused by a perceived moral violation (as described in the example of the Dutch veteran), from behavior-based dissonance (as described in the MDM)? How do persons experience moral dissonance and how can we provide solutions to alleviate dissonance caused by an is-ought problem? How often does moral dissonance lead to moral injury and what are protective factors in the process from dissonance to injury? In understanding the factors that induce dissonance, are we able to prevent it? And last but not least: what interventions at a contextual level can help counter individually felt moral distress?

Of course, there are cases in which the moral dissonance is so severe that it causes issues that can be labeled as moral injury. For these cases, treatment is needed. In therapy the patient can share the experience of a morally complex situation and the resulting feelings of shame and guilt. One form of treatment that shows a connection to the MDM is Acceptance and Commitment Therapy [ACT, (12)], which instructs the patient on the informative qualities of the moral pain. We believe the MDM can be part of the informative procedure, as it helps to define a certain type of moral dissonance and provides an easily comprehendible concept (is-ought).

In conclusion, a broader scope on what constitutes moral distress is needed to fully grasp all its influences. But even if we focus on the clinical diagnosis of moral injury, it should be recognized that it is not limited to repairing the wounds of the individual. In the end, military personnel, but also first responders and healthcare professionals are doing their work for the sake and benefit of society. Therefore, civilians should learn from and listen to their experiences of morally demanding situations (17) considering the complex and sometimes gruesome reality of these stories. Moral injury is not only a burden on the morally wounded themselves, but a matter that concerns us all.

## Data availability statement

The original contributions presented in the study are included in the article/supplementary material, further inquiries can be directed to the corresponding author.

## Author contributions

HT and BN: conception, writing, and research. All authors contributed to the article and approved the submitted version.

## Footnotes

^1^In 2019, this veteran was interviewed for a related research project that explored moral dilemmas within several professions, including military, police, prosecution, health care, and humanitarian aid work (36).
